# Diet approach before and after bariatric surgery

**DOI:** 10.1007/s11154-020-09571-8

**Published:** 2020-07-31

**Authors:** Silvia Bettini, Anna Belligoli, Roberto Fabris, Luca Busetto

**Affiliations:** 1 Luca Busetto Center for the Study and the Integrated Management of Obesity, Padova University Hospital, Padova, Italy; 2 Clinica Medica 3, Azienda Ospedaliera di Padova, Via Giustiniani 2, Padova, 35128 Italy

**Keywords:** Bariatric surgery, Nutrition, Nutritional assessment, Weight loss, Very low calories diet, Very low calories ketogenic diet, Vitamins, Reactive hypoglycaemia, Dumping syndrome

## Abstract

Bariatric surgery (BS) is today the most effective therapy for inducing long-term weight loss and for reducing comorbidity burden and mortality in patients with severe obesity. On the other hand, BS may be associated to new clinical problems, complications and side effects, in particular in the nutritional domain. Therefore, the nutritional management of the bariatric patients requires specific nutritional skills. In this paper, a brief overview of the nutritional management of the bariatric patients will be provided from pre-operative to post-operative phase. Patients with severe obesity often display micronutrient deficiencies when compared to normal weight controls. Therefore, nutritional status should be checked in every patient and correction of deficiencies attempted before surgery. At present, evidences from randomized and retrospective studies do not support the hypothesis that pre-operative weight loss could improve weight loss after BS surgery, and the insurance-mandated policy of a preoperative weight loss as a pre-requisite for admission to surgery is not supported by medical evidence. On the contrary, some studies suggest that a modest weight loss of 5–10% in the immediate preoperative period could facilitate surgery and reduce the risk of complications. Very low calories diet (VLCD) and very low calories ketogenic diets (VLCKD) are the most frequently used methods for the induction of a pre-operative weight loss today. After surgery, nutritional counselling is recommended in order to facilitate the adaptation of the eating habits to the new gastro-intestinal physiology. Nutritional deficits may arise according to the type of bariatric procedure and they should be prevented, diagnosed and eventually treated. Finally, specific nutritional problems, like dumping syndrome and reactive hypoglycaemia, can occur and should be managed largely by nutritional manipulation. In conclusion, the nutritional management of the bariatric patients requires specific nutritional skills and the intervention of experienced nutritionists and dieticians.

## Introduction

Bariatric surgery (BS) is today the most effective therapy for inducing long-term weight loss and for reducing comorbidity burden and mortality in patients with severe obesity [1]. BS has been recommended by current obesity guidelines according to body mass index (BMI) levels and associated obesity-related diseases [2].

On the other hand, BS may be associated to new clinical problems, complications and side effects, including the need to modulate the management of obesity-associated diseases according to weight loss, changes in drugs pharmacokinetic, problems in women during pregnancy, psychological difficulties in adapting to the profound changes in eating behaviour and body image, and weight regain [3]. For this reason, multidisciplinary long-term follow-up is recommended after BS and the provision of an adequate follow-up program is mandatory for bariatric centres [2].

The most important aspects in the medical management of the bariatric patient refer to nutritional management. Before BS, the nutritional status should be checked and pre-operative weight loss may be attempted. Very low calories diet (VLCD) and very low calories ketogenic diets (VLCKD) are frequently prescribed in the last months before surgery. After the procedure, nutritional counselling is important in order to facilitate the adaptation of the eating habits to the new gastro-intestinal physiology. Nutritional deficits may arise according to the type of bariatric procedure and they should be prevented and eventually treated. Finally, specific nutritional problems, like dumping syndrome and reactive hypoglycaemia, can occur and should be managed largely by nutritional manipulation. In this paper, a brief overview of the nutritional management of the bariatric patients will be provided, moving from pre-operative to post-operative phase.

## Nutritional management before BS

### Nutritional assessment before BS

Assessment of nutritional status of candidates to BS before the operation plays an important role in the post-surgical management. During the last few years, several studies demonstrated that patients with severe obesity often display micronutrient deficiencies (MDs) when compared to normal weight controls. In 2008, Asheim et al. analyzed the vitamin status of 110 patients affected by severe obesity as compared with 58 normal weight subjects: patients with obesity had significantly lower concentrations of vitamins A, B6, C, 25-hydroxyvitamin D, and lipid-standardized vitamin E [4]. Similarly, Van Rutte et al. demonstrated in 200 patients affected by severe obesity that 38% of them had low serum iron, 24% had low serum folate, 11% had low serum vit. B12 and 81% had hypovitaminosis D (55% severe deficiency with a level < 30 nmol/l) [5]. Finally, Peterson et al. demonstrated a frank deficiency of vitamin D (< 20 ng/mL) and iron (< 35 ug/dl for female and < 50 ug/dL for male) in 71.4% and 36.2% of 58 BS candidates [6].

MDs in patients with severe obesity could be attributed to a poor-quality, non-varied, high-calorie and high-fat diet. For example, excessive simple sugar, milk products or fats could lead to a deficit of vitamin B1 [7]. Moreover, iron status could be affected by adipose tissue inflammation and increased expression of the systemic iron regulatory protein hepcidin [8]. Lastly, the increased adipose mass could act as a storage site for highly lipophilic molecules, such as vitamin D, and this could explain the difference in 25(OH)D levels between people with or without obesity [9].

Assessment and correction of the nutritional status before the procedure in BS candidates is considered important for the prevention of post-bariatric MDs (see below). Indeed, Schiavo et al. recently reported that patients who received a pre-operative MDs correction did not develop new MDs in the first year after BS, whereas all patients who did not received MDs correction before BS continued to be deficient in one or more micronutrient after surgery, despite systematic post-operative supplementation [10].

### Controversies about pre-operative weight loss

Preoperative weight loss before BS is still a matter of debate. At present, most relevant guidelines do not provide any clear indication about pre-operative weight loss [11, 12]. Indeed, guidelines agreed that a period of identifiable medical management is necessary in all patients prior to BS and that it is also necessary to assess patient’s motivation and willingness to adhere to follow-up programs, but a preoperative weight loss is never mentioned nor in the indication for BS nor in the preoperative evaluation [11, 12].

The lack of clear indications for preoperative weight loss is probably related to the low evidence available on this topic. Therefore, we decided to perform a systematic analysis and we searched all articles identified as clinical trials and published in the last 10 years in PubMed with the terms “preoperative weight loss and obesity and/or bariatric surgery”. The search was conducted between June 1, 2019 and July 31, 2019 and all articles available in English were included. A total of 243 articles were retrieved: 111 duplicate articles were eliminated and further 84 were excluded because not related to the topic. Abstracts from the remaining articles where evaluated, leaving 25 full articles concerning weight loss prior surgery. After reading the full text, we concluded that only 7 out of 25 papers focused on the effects of weight loss prior to BS [13–19], and only 3 of them were randomized control trials specifically comparing patients who achieved a significant weight loss before surgery with patients who did not [13–15]. The characteristics of the three trials are reported in Table 1. Main aims of the trials were to assess if weight loss before surgery may improve the operating time [13, 15], the intra-operative complications rate [13], the surgeons perceived difficulty [13], the 30 days post-operative complications [13, 14], and the post-operative weight loss [14, 15]. The three studies presented a large heterogeneity concerning the way in which weight loss was achieved: Van Nieuwenhove et al. compared patients randomly allocated to a 2-week preoperative VLCD regimen or no preoperative dietary restriction [13], Kalarchian et al. compared patients receiving a 6-month behavioral lifestyle intervention to usual pre-surgical care [14], and Coffin et al. evaluated the impact of an intra-gastric balloon (IGB) [15]. As reported in the Table, the results are largely inconsistent for all the outcomes considered in the studies and no one single study observed a difference in post-operative weight loss between the intervention and the control arms.

**Table 1:** Results for specific outcomes in three randomised control trials specifically comparing patients who achieved a significant weight loss before surgery with patients who did not [13–15]

	Van Nieuwenhove Y et al.	Kalarchian MA et al.	Coffin B et al.
Sample size	273	143	115
Operating time	NS	/	NS
Intraoperative complications	NS	/	/
Surgeons perceived difficulty	Higher in control group vs. WL	/	/
30-days post-operative complications	Higher in control group vs. WL	NS	/
Post-operative WL	/	NS	NS

NS = not significant difference between weight loss group and control group; WL = weight loss

Apart from the few randomized trials included in our meta-analysis, most of the works on the effects of weight loss prior BS on post-surgery weight loss are retrospective studies. Giordano & Victorzon compared patients who achieved different amount of pre-operative weight loss (< 5%, > 5 to 10% and > 10%) in a retrospective study with a total sample of 548 patients: post-operative weight loss was higher in patients who achieved > 10% weight loss at 12 months, with no significant differences observed at 24 months [16]. Sherman WE et al. analyzed a cohort of 141 patients treated with sleeve gastrectomy (SG) and demonstrated that the percentage of excess BMI loss 1 year after SG was not statistically different between those who lost weight and those who gained weight before surgery [20]. McNickle & Bonomo did not found any association between pre-operative weight loss and 1-year outcomes in a cohort of 127 patients treated with a standardized 6-month medical weight loss program and a 2-week pre-operative diet with meal replacements before SG [21]. Moreover, the analysis of the data derived from the insurance mandated medical programs before BS conclude there is no evidence of any kind that insurance-mandated preoperative weight loss has any clear impact on postoperative outcomes or weight loss. For these reason, recently, the American Society for Metabolic and Bariatric Surgery published an update position statement on preoperative weight loss requirements where they conclude that “insurance-mandated preoperative weight loss is not supported by medical evidence and has not been shown to be effective for preoperative weight loss before bariatric surgery or to provide any benefit for bariatric outcomes” [22].

In summary, we can conclude that at the moment evidences from randomized and retrospective studies do not to support the hypothesis that pre-operative weight loss could improve weight loss after BS surgery. Nevertheless, no large-scale, multicenter, randomized, controlled trials have been conducted so far on this specific topic. This does not mean that patient education and dietary counselling before and after BS are not useful (see below in *Early, late and life-long Nutritional Management*).

### VLCD and VLCKD before BS may reduce surgical risk

Although BS has a low mortality rate, surgical complications (e.g., anastomotic leakage, bleeding, and infections) remain common (5–20%) and partly dependent on patient factors like age, sex, and comorbidity [23]. Laparoscopic surgery in patients with severe obesity is challenging because of the thickness of the abdominal wall, intra-abdominal obesity, possible mesenteric thickening, and hepatomegaly [24]. The presence of visceral fat can increase the complexity and risk in patients undergoing any type of abdominal surgery [25]. Thickened abdominal walls may limit precise surgical movements during laparoscopy, and intra-abdominal obesity can limit visibility during surgical procedures. Nonalcoholic fatty liver disease (NAFLD) is a condition frequently complicating obesity that can lead to an increase in liver fat infiltration, mainly in the left lobe, making the liver brittle and more susceptible to injury and bleeding. During laparoscopic bariatric surgery, hepatomegaly and visceral fat in the left upper quadrant may limit preliminary exposure of the surgical field [26–28] and may increase the conversion rate and operative time [29]. The overall conversion rate in Roux-en-Y gastric bypass (RYGB) is approximately 4%, and an enlarged liver is responsible for approximately 50% of the conversions [29].

In this contest, a modest weight loss of 5–10% in the immediate preoperative period has been suggested as a mean to facilitate surgery and reduce the risk of complications. Preoperative weight loss can be obtained with several regimens, such as low-calorie diets (LCD) (800–1200 kcal/day), very low-calorie diets (VLCD) (600 kcal/day), or a hypocaloric diet combined with IGB placement, and the question of which method provides the best results in terms of weight loss and patients’ compliance, tolerance, and acceptance is still under debate. The use of IGB prior to gastric banding surgery was described to significantly decrease the conversion rate and intra-operative complications in a matched case-control study [30]. A pre-operative VLCD may also induce a significant weight loss before BS, being faster, cheaper, and with fewer side effects than IGB [31]. Preoperative weight loss by means of a VLCD has been reported to reduce liver size and intra-abdominal fat mass, blood loss, short-term complications as well as operation time and length of hospital stay [13, 26, 27, 32, 33]. However, a systematic review confirmed that VLCD led to a significant weight loss (− 2.8 to − 14.8 kg) and liver size reduction (5–20% of the initial volume), but did not found a reduction in peri-operative complications [34]. Andrianzen Vargas et al. showed that LCD is insufficient in 60% of cases to achieve the intended 10% weight loss, while VLCD is able to achieve it in practically all patients [31]. However, a more recent study comparing the effect of VLCD and LCD before surgery showed that, although VLCD was more effective in reducing total body weight (5.8 vs. 4.2%), there were no differences in terms of liver volume reduction, with both diets having similar effects on biochemical parameters, rate of surgical complications, and hospital length stay [35].

More recently, very low-calorie ketogenic diet (VLCKD) has been proposed as a new effective and safe method for achieving effective preoperative weight loss. Previous studies reported that VLCKDs are effective for weight loss and safe in non-surgical contexts [36]. On the other hand, it must be considered that any very low-calorie regimen drives a catabolic state and an increased oxidative stress that may have a negative impact on surgical outcomes. In addition, the ketogenic diet, based only on a protein substrate, may induce an adaptive response in several organs, with physiological modifications potentially unsafe in the perioperative period. To date only few studies addressed the role of VLCKD immediately before BS, and the available data are actually scarce. Leonetti et al. evaluated in an uncontrolled study the compliance, safety, and effectiveness of a sequential regimen (VLCKD for 10 days, followed by a VLCD for 10 days, and then a LCD for 10 days) in patients with obesity scheduled for BS. The study showed an adequate short-term reduction of body weight and waist circumference, without dangerous alteration in renal, hepatic, and metabolic functions. The weight loss was similar to that obtained with a VLCD and better than reported for LCD [37]. A similar 30-day sequential preoperative regimen was used in another uncontrolled study showing a significant reduction in weight, waist circumference and visceral fat, and an improvement in several clinical parameters, including glycemic and lipid profiles. Moreover, a mean 30% reduction in liver volume was also observed [38]. Finally, in a third non randomized study, patients were treated with either VLCKD or VLCD for 3 weeks prior to BS. Weight loss was not significantly better in the VLCKD than in the VLCD group, but VLCKD had better results on surgical outcomes, influencing drainage output, post-operative hemoglobin levels, and hospital stay. However, no data on the reduction of liver and visceral fat volumes were obtained in this study [39].

In conclusion, there is a general agreement on the beneficial effects of a modest weight loss in the immediate pre-surgical period on the surgical and anesthesiological risks. Efficacy of VLCD regimens and IGB as bridging therapy before BS is consolidated in literature, while the role of VLCKD is arising in importance, but still under debate in the pre-operative period. Large randomized studies addressing these issues are needed, particularly aimed to accurately measure the effects on liver and visceral fat volumes changes.

## Nutritional approach after BS

### Characteristics and nutritional impact of the bariatric procedures

**Fig. 1:**
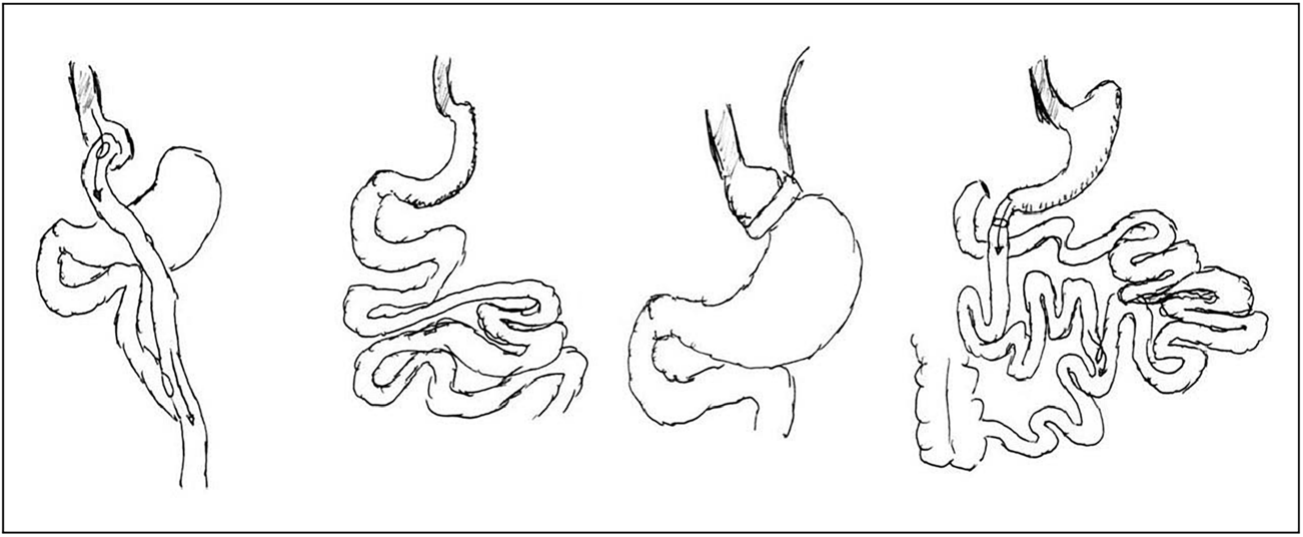
A graphical representation of the anatomical features of the more frequently adopted bariatric procedures. From left to right: Roux-en-Y gastric bypass, sleeve gastrectomy, adjustable gastric banding, and biliopancreatic diversion with duodenal switch. Adapted from: Catoi AF, Parvu A, Muresan A, Busetto L. Metabolic mechanisms in obesity and type 2 diabetes: insights from bariatric/metabolic surgery. Obesity Facts 2015;8:350 − 63

Bariatric procedures have been classically distinguished in restrictive, mixed or malabsorptive according to the anatomy and the principal mechanism of action. This classification has been recently criticized in an era in which the metabolic mechanisms of action of BS received much more attention than the anatomical or functional ones. However, from the nutritional point of view, this classification remains valid, since the impacts of BS on the nutritional status are mostly linked to the reduction of the volume of the stomach and to the reduced absorption of nutrients. A graphical representation of the anatomical features of the more frequently adopted bariatric procedures is provided in Fig. 1.

### Early, late and life-long Nutritional Management

As shown before, most bariatric procedures include the reduction of the volume of the stomach and/or the creation of a small gastric pouch. Because of the small volume and the post-operative gastric oedema, the ingestion of solid foods in the first days after surgery is very difficult or impossible. Therefore, in order to avoid or minimize regurgitation and vomiting, most post-operative nutritional protocols suggest a liquid or very soft diet in the first days after surgery and a very gradual increase in food consistency in the first post-operative weeks [40]. Usually, a low-sugar clear liquid diet is initiated within 24 h of surgery and patients are then instructed to gradually and progressively change the food consistency, moving from clear liquids to soft or creamy foods and then to solid chewable items over a period of 2–4 weeks [11, 40, 41]. Before discharge, patients should receive specific counselling by an experienced bariatric dietician about this postoperative meal progression [3, 11].

After the end of the post-operative diet and thereafter, patients should receive periodic counselling by a registered dietician about long-term dietary modifications, in order to maximize the results of the bariatric procedure and reduce the risk of late weight regain. The focus of dietary counselling should be the adaptation of patients eating behaviour to the surgical procedure and the general qualitative aspects of a healthy nutrient dense diet [3, 11, 40, 41]. Sarwer et al. randomised 84 patients treated with BS to receive either dietary counselling or standard care for the first 4 months after surgery [42]. Weight loss was slightly, but not significantly elevated in the dietary counselling group. However, patients in the dietary counselling group reported significant positive changes in several eating behaviours believed to be important to successful long-term weight maintenance [42]. The addition of some behavioural component to standard dietary counselling after BS is also supported by some randomised, prospective and retrospective study. Despite limitations due to the small and heterogeneous samples of studies, a meta-analysis conducted by Rudolph & Hilbert suggest greater weight loss in patients with behavioural lifestyle interventions compared with control groups in five randomised controlled trials [43].

### Protein Intake and Protein Supplementation

Weight loss in the first months after BS is rapid and it may be associated to a significant unintended loss of fat-free mass and muscle mass [44]. Sufficient protein intake is considered protective against the loss of lean body mass during rapid weight loss, but protein intake is frequently and substantially reduced after BS, particularly in the first months after the procedure, mostly because of the gastric intolerance to protein-rich foods [4]. Current guidelines recommend a minimal target for protein intake after BS of 60 g/day and up to 1.5 g/kg ideal body weight per day [3], but higher amounts of protein intake (up to 2.1 g/kg ideal body weight per day) may be required in individual cases [11]. Considering the difficulties in reaching these demanding targets with natural foods only, the use of liquid protein supplements (30 g/d) is suggested as a mean for facilitating adequate protein intake in the first months after BS [3].

Current recommendations regarding protein intake and supplementation after BS are based on the results of a prospective one-year observational study showing an inverse relationship between protein intake and lean tissue loss, as a percent of total weight loss, in 50 patients treated by RYGB (25 patients) or SG (25 patients) analyzed with dual energy X-ray absorptiometry (DEXA) [45]. In particular, a protein intake > 60 g/day was associated with lower fat-free mass loss [45]. A small randomized trial recently questioned the effectiveness of protein supplementation in tempering lean body tissues loss [46]. In the trial, Oppert et al. randomly assigned 76 patients to usual care after BS, usual care and additional whey protein supplementation (48 g/day), or usual care and protein supplementation and supervised strength training (1 h three times per week). Loss in lean body mass did not differ between groups, whereas an increase in muscle strength was observed only in the protein plus exercise group [46]. The results of this small trial probably did not have the power to modify previous prudent recommendations, but they emphasizes the importance of including strength training in the types of physical activities recommended after BS [3].

### Micro-nutrients Deficiencies and Supplementation

Vitamins and mineral deficiencies are relatively common after BS. The anatomical characteristics and the mechanisms of action of the various procedures dictate their frequency and severity. Nutritional deficiencies are uncommon after purely gastric restrictive procedures not altering intestinal continuity and normal digestive processes, but more common after surgical procedures inducing some degree of malabsorption [3, 11]. A brief overview of the major vitamins and minerals deficiencies occurring after BS, their clinical manifestations, and the estimated frequency according to the bariatric procedure is provided in Table 2. More detailed information can be found in several guidelines for nutritional management after BS [3, 11, 40, 41].

**Table 2:** Major vitamins and minerals deficiencies after bariatric surgery: clinical manifestations and estimated frequency according to the bariatric procedure. (Adapted from Reference [3]

Deficiency	Key Clinical Manifestations	Procedure-related frequency
Iron	Microcytic Anemia	AGB + SG ++ RYGB, BPD, BPD/DS +++
Vitamin B12	Megaloblastic Anemia Neurologic abnormalities	SG, RYGB, BPD, BPD/DS ++
Vitamin D (and Calcium)	Bone demineralization Increased risk of fractures	RYGB, ++ BPD, BPD/DS +++
Vitamin A	Ocular xerosis Night blindness symptoms	BPD, BPD/DS +++
Vitamin E	Anemia, Ophthalmoplegia Peripheral neuropathy	BPD, BPD/DS +++
Vitamin K	Easy bleeding	BPD, BPD/DS +

AGB: adjustable gastric banding; SG: sleeve gastrectomy; RYGB: gastric bypass; BPD: biliopancreatic diversion; BPD/DS: biliopancreatic diversion with duodenal switch

Prevention, detection and treatment of vitamins and minerals deficiencies represent cornerstones of long-term follow-up after BS. Routine daily multivitamin and mineral supplementation should be prescribed in every patients after BS according to the type of procedure and current guidelines [3, 11, 40, 41]. However, routine supplementation does not confer an absolute protection from deficiencies over time, because of individual variations in micro-nutrients absorption, nutritional requirements and compliance. Therefore, periodic laboratory routine surveillance for nutritional deficiencies is recommended and supplementation should be individualized accordingly, with patients with demonstrated micronutrient insufficiencies treated with the respective micronutrient [3, 11].

## Specific nutritional problems after BS

### Hypoglycemia and dumping syndrome

Although BS has resulted in numerous health benefits, including diabetes remission, it is also associated with a still underestimated complication: post-prandial reactive hypoglycemia. True prevalence of BS-related hypoglycemia is unknown, partly due to lack of consensus in defining and diagnosing this condition [47–49]. Reactive hypoglycemia is generally considered to be more common after RYGB than SG, despite the recent report of a high hypoglycemia rate 1-year after SG [50]. A 1-year randomized trial comparing the effect of SG with that of RYGB showed that hypoglycemic events measured with continuous glucose monitoring was not different between the two procedures (29% vs. 14%) [51]. However, RYGB was associated with more severe hypoglycemic episodes and RYGB patients had an overall larger number of reactive hypoglycemic episodes than SG. This fact was explained by the postprandial hyperinsulinemia observed after RYGB, inappropriately high despite glucose levels, and not by gastric empting, described to be equally accelerated after both RYGB and SG [52]. A study comparing matched controls to RYGB surgical patients with and without symptomatic hypoglycemia, shown that glucagon-like peptide 1 (GLP-1) had effect on insulin secretion only in RYGB patients, both symptomatic and asymptomatic. Indeed, GLP-1 levels were increased by 10-fold after meal in patients undergoing RYGB and the rate of glucose appearance in the circulation was higher in symptomatic patients compared with asymptomatic patients [53]. RYGB patients are often unaware of low glycemic levels as a progressive adaptation to hypoglycemia. Furthermore, it was described that RYGB reduces symptoms and hormonal responses to hypoglycemia [54].

Diagnosis of post BS hypoglycemia requires the compresence of neuro-glycopenic symptoms (such as flushing, weakness, loss of consciousness), blood glucose concentration less than 3.0 mmol/L (54 mg/dL), and symptoms relief by carbohydrates, according to the Whipple’s triad [55, 56]. Available provocation tests are the oral glucose tolerance test (OGTT) and the mixed meal tolerance test. Although the latest test should be considered the best provocative test to diagnose post-prandial reactive hypoglycemia, there is no currently an accepted standard meal test.

Dumping symptoms, caused by the release of gut hormones and the rapid entry of water into the intestinal lumen, are classified on the time of development after eating as early and late symptoms. Early dumping represents the most common type, reaching 40% after RYGB and SG [57, 58], while late dumping is observed in only 25% of patients [59]. Early symptoms are predominantly vasomotor (such as palpitation, flushing and syncope) and gastrointestinal symptoms (abdominal pain, diarrhea, bloating and nausea) and occur within 15 min after meal, and late symptoms (tremor, perspiration, aggression, fatigue, weakness, confusion, hunger and syncope) occur in 1–3 h after eating when glycaemia reaches nadir levels. Two questionnaires have been established for the identification of dumping syndrome, the Sigstad’s score [60] and the Arts’ dumping questionnaire [61]. Recently, Salehi et al. proposed a possible schematic approach to post-prandial hypoglycemia in patients underwent RYGB and they suggested that post prandial hypoglycemia can be diagnosed when the following criteria are met, and other causes of hypoglycemia have been ruled out: (1) history of postprandial neuroglycopenia occurring 1 to 3 h after meals in a patient with history of BS at least 6 to 12 months before symptom onset, (2) documented hypoglycemia (venous glucose, 54 mg/dL) at time of neuroglycopenic symptoms, with resolution of symptoms with treatment to raise glucose, (3) no hypoglycemia after a prolonged fast of at least 12 h [62].

The terms hypoglycemia and late dumping syndrome have often been used interchangeably. Hypoglycemia after BS is exclusively post-prandial and many bariatric patients have postprandial autonomic symptoms, such as lightheadedness, palpitations and fatigue, which may represent manifestations of the dumping syndrome but also overlap significantly with the autonomic symptoms of hypoglycemia. To differentiate between symptoms related to post-RYGB hypoglycemia or late dumping, we can use the time of onset from surgery, with dumping usually developing shortly after RYGB (< 3 months) and hypoglycemia generally manifesting later [53, 63]. In a cross-sectional study with and without documented post-RYGB hypoglycemia, it was demonstrated that a history of dumping syndrome did not increase the likelihood of hypoglycemia during meal studies [53], suggesting that dumping and hypoglycemia may share the common underlying mechanism (rapid nutrient passage from stomach pouch to the gut), but they manifest different aspects of deregulated gut physiology.

Treatment approaches for post-bariatric hypoglycemia and dumping syndrome include dietary modifications, pharmacologic interventions and, rarely, surgical re-intervention or continuous tube feeding. From a nutritional point of view, smaller and more frequent meals (around six per day) are recommended and intake of fluids should be delayed by at least 30 min after meal [3, 59]. Rapidly absorbable carbohydrates and alcoholic beverages should be avoided, while intake of high-fibers, high-protein foods is suggested. Lying down after a meal for 30 min may further delay emptying of the stomach.

### Food Intolerance and changes in food-preferences

Studies reported marked reductions in hunger and more postprandial fullness after BS, resulting in a reduced meal size without compensatory increase in meal frequency [64–66]. Several factors have been related to the reduction in appetite and energy intake. Alterations in gastrointestinal anatomy result in neurological and physiological changes affecting hypothalamic signaling and gut hormones [66, 67]. Furthermore, BS not only increases satiety but also affects food preferences, including increased acuity to sweet taste, and decreased hedonic evaluation of sweet and fatty foods [68]. GLP-1 is released in the gut from the sweet-sensitive taste cells after stimulation with sweet compounds, suggesting that this gut peptide might contribute to the increased sweet taste sensitivity after BS [69]. It was shown that meals of solid texture and smaller meals were better tolerated and generated less hypoglycemia than large and/or liquid meals after RYGB [70]. Faulconbridge et al. demonstrated that the neural response of high-calorie foods 6 months after RYGB and SG decreased significantly and remained stable in the control group [71].

Food intolerance after BS could be explained by the “conditioned taste avoidance” [69], which refers to a conscious adjustment to food that is still palatable but has caused adverse reactions when larger quantities are consumed. Early dumping, as described above, may promote post-prandial discomfort and lead to a conditional aversion changes in eating patterns after SG, especially in sweets [57]. However, objective methods for assessing eating behavior in humans are limited by a complex set of cultural and psycho-social factors and conclusions are challenged by the use of self-reported data. In contrast, Nielsen et al. established that total energy intake decreased 6 and 18 months after BS, but there were no changes in food preference compared with before surgery [72]. Furthermore, at 6 months after BS the increased preference for low-fat savory foods compared with before surgery could indicate that patients were more aware of the nutritional recommendations in the early postoperative period and this awareness fell with time after BS [72].

## Conclusions

BS induces significant and long-lasting changes in nutritional habits and eating behaviour. The anatomical and functional modifications of the gastro-intestinal tract produced by BS always require the adaptation of patients eating behaviour to the new gastro-intestinal physiology, and procedure-specific nutritional problems and symptoms may occur. Adequate nutritional management is also important in the pre-operative phase. The nutritional management of the bariatric patients requires therefore specific nutritional skills and the intervention of experienced nutritionists and dieticians. Detailed guidelines for post-operative bariatric nutritional management have been published and recently updated [3, 40, 41].
